# Real World Experiences: Pirfenidone and Nintedanib are Effective and Well Tolerated Treatments for Idiopathic Pulmonary Fibrosis

**DOI:** 10.3390/jcm5090078

**Published:** 2016-09-02

**Authors:** Gareth Hughes, Hannah Toellner, Helen Morris, Colm Leonard, Nazia Chaudhuri

**Affiliations:** 1 North West Interstitial Lung Disease Unit, University Hospital of South Manchester, Wythenshawe Hospital, Southmoor Road, Wythenshawe, Manchester M29 9LT, UK; gareth.e.hughes@doctors.org.uk (G.H.); helen.morris4@nhs.net (H.M.); Colm.Leonard@UHSM.NHS.UK (C.L.); 2 Manchester Academic Health Science Centre, The University of Manchester, Oxford Road, Manchester M13 9PL, UK; 3 Manchester Medical School, The University of Manchester, Oxford Road, Manchester M13 9PL, UK; hannah.toellner@student.manchester.ac.uk

**Keywords:** pirfenidone, nintedanib, IPF, Idiopathic Pulmonary Fibrosis

## Abstract

Idiopathic Pulmonary Fibrosis (IPF) now has two licensed treatments available. Pirfenidone was the first drug to be licensed and approved for use, followed by nintedanib. We set out our real world experience with these agents in terms of their adverse events profile outside the restrictions of a clinical trial. We have demonstrated in the real world setting, that side effects are common and predominantly gastrointestinal with both therapies. Our study shows that the side effects can be effectively managed in the majority of patients with an acceptable discontinuation rate similar to that seen in the clinical trials. These findings are compelling despite the fact that the patients in our study are older, have severer disease as depicted by baseline lung function and more co-morbidities. Our data provides ongoing evidence of the safety and tolerability of both pirfenidone and nintedanib in patients who would not have met the rigorous criteria to be included in a clinical trial. Both these agents are effective in the management of IPF and slow the progression of this debilitating life limiting condition.

## 1. Introduction

Idiopathic Pulmonary Fibrosis (IPF) is a debilitating life limiting condition with a median survival ranging from 2.5 to 3.5 years [1]. Current treatment strategies advocate a multidisciplinary approach to diagnosis and management with a holistic approach encompassing pulmonary rehabilitation, oxygen therapy, palliation and anti-fibrotic therapies [2,3]. Pirfenidone, the first drug to be licensed, was approved for use by the National Institute for Health and Care Excellence (NICE) in 2013 [4]. Nintedanib, a tyrosine kinase inhibitor, has also recently been licenced and approved by NICE for the management of IPF in 2016 [5].

Pirfenidone has both anti-inflammatory and anti-fibrotic effects [4]. Pooled data from the two CAPACITY [6] phase three randomised controlled trials (RCT) demonstrated a 2.5% absolute reduction (pirfenidone −8.5% mean change and Placebo −11%) and 22.8% relative reduction in the decline of FVC as well as a 26% improvement in progression free survival. Data was pooled as one of the CAPACITY studies did not meet the primary endpoint. Following CAPACITY results a European licence was granted but the United States Food and Drug Administration (US FDA) requested further evidence of benefit. The ASCEND [7] study was designed to assess whether pirfenidone reduced disease progression in patients with IPF. Learning from the CAPACITY studies, the ASCEND study design was specifically adjusted to focus on patients most likely to benefit from pirfenidone treatment i.e., those with no pre-existing emphysema. ASCEND robustly confirmed the findings of the pooled CAPACITY [6] data and demonstrated a 47.9% relative reduction in the decline of FVC and benefit in terms of reduced decline in six-minute walk test. A pre-specified pooled analysis of both CAPACITY and ASCEND mortality data, as requested by the FDA, demonstrated a statistically significant improvement in all cause and IPF related mortality. In both the CAPACITY [6] and ASCEND [7] studies similar side-effect profiles were found. Patients experience an increase in gastrointestinal events (nausea, dyspepsia, vomiting and anorexia) and skin disorders (rash and photosensitivity) however symptoms were generally mild or moderate and without any long term sequelae. In the ASCEND [7] study adverse events led to a discontinuation rate of 14.4% in the pirfenidone group versus 10.8% in the placebo group. These findings were comparable with the CAPACITY [6] trials that showed a 15% discontinuation rate in the pirfenidone group versus 9% in the placebo group. Both these studies suggested a favourable risk benefit profile and a clear benefit in the treatment of IPF.

Nintedanib targets specific intracellular tyrosine kinase enzymes, involved in cell signaling cascades which are important in the development of IPF [8]. An early phase 2 trial: ‘To Improve IPF with BIBF’ [9] identified that a dose of 150 mg twice a day over a 12 month period slowed the decline in FVC in IPF patients [9]. There was a 68.4% drop in the rate of annual FVC decline compared to placebo in this patient group. Nintedanib also decreased the frequency of acute exacerbations (2.4 vs. 15.7 per 100 patient-years, *p* = 0.02) [9]. Two further phase 2 RCTs, INPULSIS 1 and 2 [10] were carried out over 52 weeks with a total number of 1066 patients. Patients were allocated in a 3:2 ratio to be given nintedanib 150 mg twice daily or placebo. The results from both studies provided stronger evidence that nintedanib 150 mg twice daily slows FVC decline compared to placebo. The INPULSIS-1 trial showed that the annual rate of change in FVC was −114.7 mL with nintedanib compared to −239.9 mL with placebo (*p* < 0.001) and −113.6 mL to −207.3 mL in INPULSIS-2 (*p* < 0.001). However, the effect on time to first acute exacerbation was inconsistent across both studies. Neither trial was powered to assess a mortality effect [10].

The majority of the most common reported adverse events (AEs) identified in both studies were classified as mild to moderate in nature. Diarrhoea occurred frequently in the nintedanib treatment group (INPULSIS-1: 61.5% vs. 18.6% placebo, INPULSIS-2: 63.2% vs. 18.3%), followed by nausea (22.7% vs. 5.9%, 26.1% vs. 7.3%), nasopharyngitis (12.6% vs. 16.7%, 14.6% vs. 15.5%) and cough (15.2% vs. 12.7%, 11.6% vs. 14.2%). Elevated liver enzymes were also more commonly identified in the patients receiving nintedanib. The overall proportion of patients with serious AEs was comparable in both treatment and placebo groups [10]. Nintedanib was completely discontinued in 25.2% in patients in the treatment group vs. 17.6% in the placebo group in INPULSIS-1. In INPULSIS-2 23.7% patients discontinued their treatment compared to 20.1% of the placebo group. Discontinuations were most often related to an AE. However, less than 5% of patients in both studies suffering from diarrhoea discontinued their treatment. An average dose intensity of greater than 90% in the nintedanib groups emphasises that these AEs were managed acceptably and tolerated by the majority of patients, thus preventing total discontinuation of the drug. Patients were able to reduce their dose from 150 mg to 100 mg or temporarily cease treatment during an AE, before returning to the full dose once the symptoms had resolved [10]. Nintedanib also targets vascular endothelial growth factor receptor (VEGF) and platelet derived growth factor receptor (PDGFR). Reassuringly in these trials there was no increased signal regarding cardiovascular or bleeding complications with nintedanib therapy. These studies show that nintedanib has an acceptable AE profile and is effective in reducing progression of IPF.

We have previously published our early clinical experience of pirfenidone use in patients with IPF [11]. We and others have shown a favourable adverse event profile and treatment compliance during the early phase of treatment [11,12,13]. Here we share real world experience of pirfenidone in patients with IPF spanning an extended four and half year period. We also share our early clinical experiences with nintedanib therapy spanning 18 months.

## 2. Methods

This is a single centre retrospective observational study. We reviewed the data for 351 patients initially involved in the manufacturer funded Named Patient Programme (NPP) for pirfenidone between September 2011 and April 2013 (*n* = 48) and those that were subsequently prescribed pirfenidone after NICE approval in April 2013 (*n* = 303). Inclusion criteria included a multidisciplinary team (MDT) diagnosis of IPF and FVC greater than 50% and/or carbon monoxide diffusing capacity (DLCO) greater than 35% predicted for the NPP and FVC 50%–80% for those managed after NICE approval.

We also present the data for 124 patients, who commenced treatment with nintedanib from December 2014 to June 2016 as part of a manufacturer funded patient in need (PIN) programme prior to NICE approval. The inclusion criteria included all patients who had an MDT diagnosis of IPF and who were not eligible for pirfenidone by NICE criteria due to an FVC less than 50% or greater than 80% or who were unable to tolerate pirfenidone side effects.

All patients had baseline pulmonary function tests (PFTs), full blood count (FBC), urea and electrolyte (U+E) and liver function tests (LFTs) prior to commencing therapy. Both pirfenidone and nintedanib were prescribed as per manufacturer’s recommendations. Pirfenidone was titrated to a dose of 3 × 267 mg capsules three times daily (total 2403 mg/day) as tolerated. Nintedanib was prescribed at 150 mg twice a day. Patients were reviewed clinically on a monthly basis by a health care professional for three months then monthly thereafter for pirfenidone and monthly for the first six months with nintedanib, and then three monthly thereafter, or earlier according to clinical need. Each clinical review involved confirmation and documentation of treatment dose, development of AEs, strategies to manage AEs, documentation of dose interruptions and any chest infections or hospital admissions in the preceding months. FBC, U+E and LFTs were also monitored at each review and PFTs were performed at 6 monthly intervals. For patients with pre-treatment PFTs we compared decline in FVC before and after treatment.

Data was analysed using GraphPad Prism v5. Where indicated data was compared using One way ANOVA and Dunnett’s post test or unpaired *t*-test.

## 3. Results

### 3.1. Pirfenidone Data: Patient Demographics

Our pirfenidone-treated cohort had an average age of 70 years (range 47–91) with 81% of those being male. Lung function showed a pre-treatment average FVC of 69% (*n* = 346, range 37%–146%) and DLCO 42% (*n* = 258, range 14%–112%). Shuttle walk data was available for 21% of patients with an average of 358 m (range 20–1020 m). The majority of patients had been ex or current smokers (*n* = 229, 65% and *n* = 83% respectively) with 16% (*n* = 56) having evidence of emphysema on CT scan. Twenty six percent (*n* = 90) of patients were on either ambulatory or long-term oxygen therapy.

### 3.2. Gastrointestinal Adverse Effects Are Common With Pirfenidone Therapy

**Figure 1:**
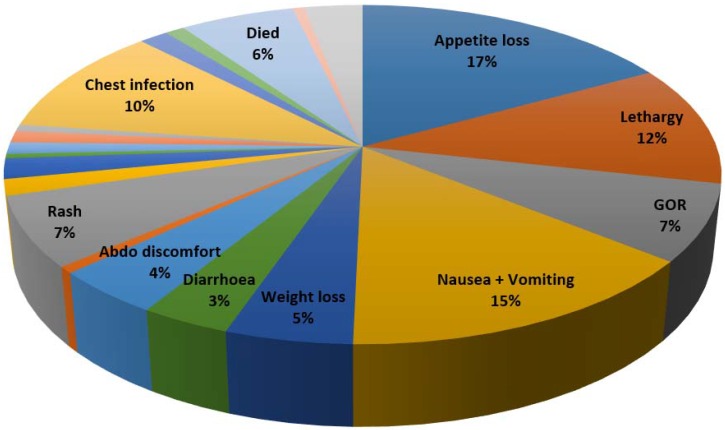
Frequency of Adverse Events with Pirfenidone.

There were a total of 973 AEs in 264 patients (average of 3.6 AEs per patient). Seventy six (22%) patients experienced no AEs throughout their treatment and monitoring period. Only a minority of patients experienced 1 AE (*n* = 49, 14%) with the majority experiencing more than 2 AEs. In total 215 (61%) patients had more than 2 adverse events whilst 74 (21%) had five or more. Seventy five percent of AEs were gastrointestinal with loss of appetite (17%), nausea (15%) and lethargy (12%) being the most frequent (Figure 1). Chest infection (10%), Rash (7%) and Gastro-oesophageal reflux (7%) were also prevalent (Table 1).

The Majority of AEs with pirfenidone therapy are manageable by dose reduction and slower dose titrations.

**Figure 2:**
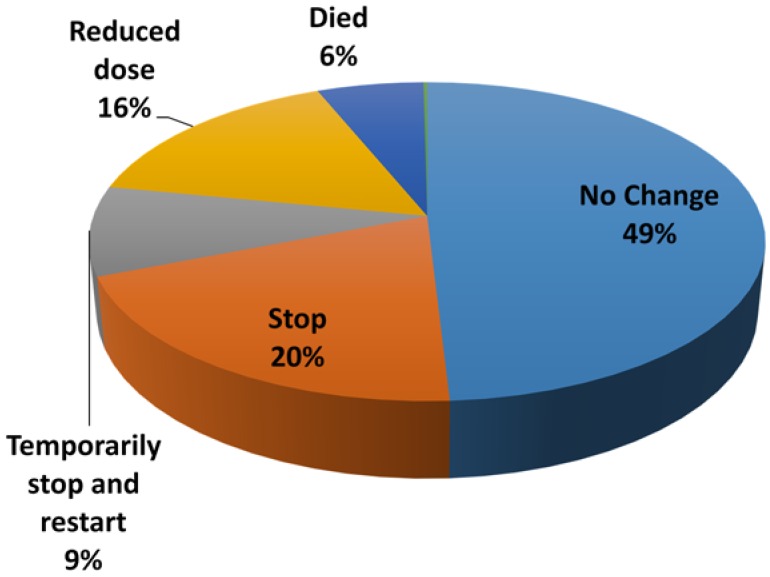
Impact of AE on treatment with Pirfenidone.

The majority of AEs resulted in no change in pirfenidone treatment (*n* = 486, 49%). One hundred and fifty six AEs resulted in reduction of pirfenidone dose (20%). In 90 AEs pirfenidone was temporarily stopped and restarted (9%) and in 197 AEs pirfenidone was permanently discontinued (20%) (Figure 2).

The highest number of treatment terminations occurred with symptoms of appetite loss and nausea/vomiting with 28 and 22 discontinuations respectively. In total 30% of pirfenidone patients had dose modifications due to their AEs, 29% discontinued treatment permanently and there was a 20% mortality over the four and a half year period.

**Figure 3:**
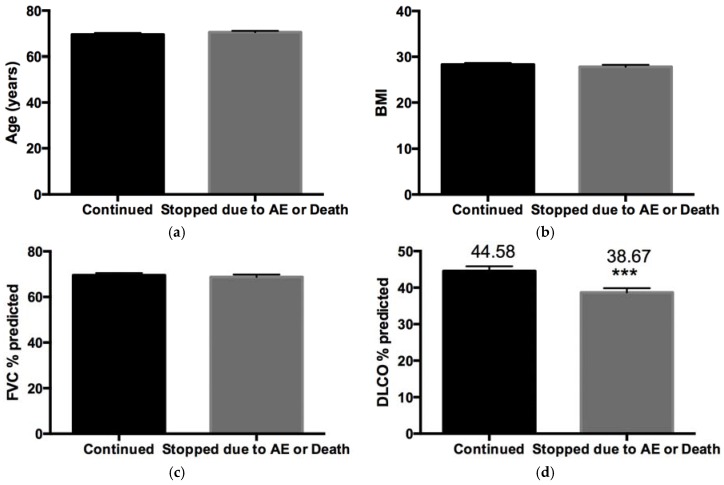

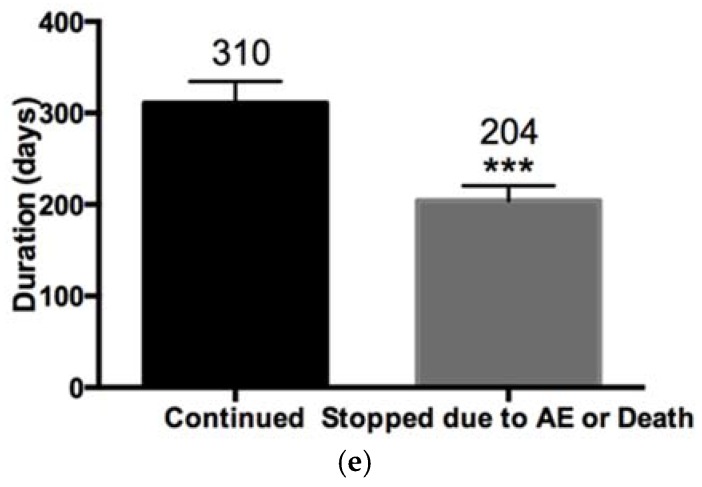
Impact of age, BMI, FVC, DLCO and duration of treatment on discontinuations due to AEs (Where *** denotes *p* < 0.001)

Patients who stopped treatment due to AEs persevered with treatment for a mean of 204 days (Figure 3e), had more severe disease (*p* < 0.05) as depicted by baseline DLCO (Figure 3d), but no difference in age, body mass index (BMI) or baseline FVC (Figure 3a–c).

### 3.3. Pirfenidone Demonstrates a Reduction in the Decline in FVC over Time

**Figure 4:**
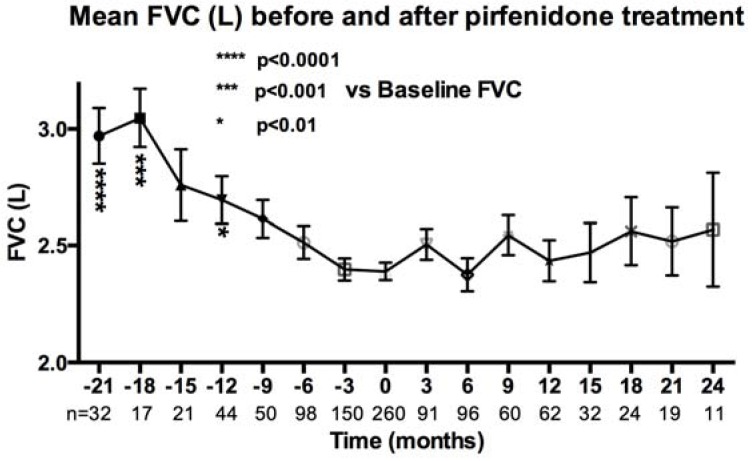
Reduction in decline of mean FVC before and after pirfenidone treatment.

**Figure 5:**
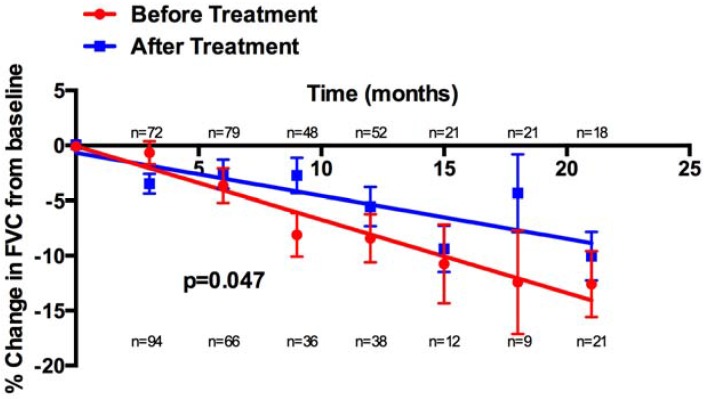
Percentage change in FVC from baseline before and after treatment with pirfenidone.

After initiation of pirfenidone treatment there was a reduction in the rate of decline of mean FVC (Figure 4) and percentage change of FVC from baseline (Figure 5).

### 3.4. Nintedanib Data: Patient Demographics

Our nintedanib cohort had an average age of 71 ± 8.5 years (range 50–89), and 73% were male. The average length of treatment for 113 patients was 258 days ± 138 (range 12–492). Pre-treatment average FVC was 81% predicted (*n* = 122, range 35%–120%) and DLCO was 44% predicted (*n* = 82, range 13%–78%). Shuttle walk data was available for 32% of patients with a most recent average of 397 m (range: 90–900) and a pre- and post- walk average saturation value of 96% and 87% respectively.

The majority of patients had been ex (*n* = 83, 67%) or current smokers (*n* = 3, 2%), in contrast to 35 who had never smoked (28%) and 3 unknown (2%). The average pack year smoking history for current and ex-smoking patients, where this information was available, was 33 pack years (*n* = 75). 35% of patients were using ambulatory and/or long term oxygen therapy (*n* = 43).

The reasons for commencing nintedanib were an FVC greater than 80% in 68 (55%) patients, an FVC less than 50% predicted in 12 (10%) patients and intolerable AEs with pirfenidone in the majority of the remaining patients (*n* = 33, 27%).

### 3.5. Adverse Events Are Common and Predominantly Gastrointestinal with Nintedanib

A total of 668 AEs were reported in 119 patients, (5.4 AEs per individual patient). 5 patients had no follow up data and no AEs recorded. A significant majority of the remaining patients had reported at least two or more AEs (82%, *n* = 102) and 54% had 5 or more AEs (*n* = 67). When each AE was not counted separately at follow up, a total of 442 AEs were reported in 119 patients, corresponding to 3.6 AEs per patient.

**Figure 6:**
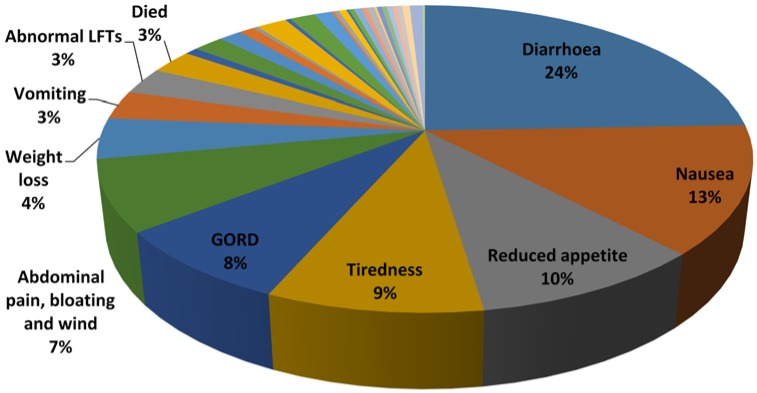
Frequency of Adverse Events with Nintedanib.

The most frequent AEs were diarrhoea (24%), followed by nausea (13%), reduced appetite (10%), tiredness (9%) and gastro-oesophageal reflux (GORD) (8%). Less common AEs included acute kidney injury (AKI) (1%, *n* = 4), myocardial infarction (MI) (<1%, *n* = 2), haemoptysis (<1%, *n* = 3), pulmonary embolism (P.E) (<1%, *n* = 1) and haematemesis (<1%, *n* = 1) (Figure 6, Table 2).

### 3.6. AEs with Nintedanib Are Well Tolerated

At the end of the monitoring period, treatment was discontinued in 26% of patients (*n* = 32). 19% stopped permanently (*n* = 23) and 7% paused treatment temporarily (*n* = 9). 45% of patients continued treatment at the full dose (*n* = 56), 15% were taking a reduced dose (*n* = 19) and 14% had died (*n* = 17).

Overall, 71 patients experienced at least 1 AE of diarrhoea (57%). There were a total of 163 diarrhoea AEs in this group of patients. No change in treatment was reported for 71% of these AEs (*n* = 115) and 22% led to a reduction in dose of nintedanib (*n* = 36). 7% of all AEs of diarrhoea resulted in discontinuation of treatment either on a temporary or permanent basis (*n* = 12).

## 4. Discussion

Here we demonstrate our real world experiences of patients with Idiopathic Pulmonary Fibrosis being treated with anti-fibrotic therapies in a single UK center. We have been actively involved with clinical trial recruitment and early patient access schemes for IPF and as a result have gained over four years experience and clinical data of pirfenidone treatment in IPF and 18 months experience with nintedanib.

Our data shows favourable outcomes with respect to the safety of both pirfenidone and nintedanib. We demonstrate that the majority of patients experience two or more adverse events during pirfenidone and five or more during nintedanib treatment. The majority of these events are gastrointestinal in nature for both anti-fibrotic agents with appetite loss, nausea and lethargy being the commonest with pirfenidone and diarrhoea, nausea and reduced appetite with nintedanib. Skin-related events were specific to pirfenidone. The type of adverse events in the real world are similar to that demonstrated in the CAPACITY [6], ASCEND [7] and INPULSIS [10] studies. Clinical trials are more robust and vigorous in their monitoring of AEs and this may reflect why the frequency of reported events are lower in our real world data. Reassuringly, in our 18 month experience with nintedanib we did not experience any signals with respect to increased cardiovascular morbidity or bleeding risk. Only a very small number of our patients (*n* = 2) were also treated with anticoagulation. Further long term data is therefore required in patients prescribed nintedanib and anticoagulants or dual antiplatelet therapy.

The majority of AEs with pirfenidone (80%) were tolerated or managed by dose reduction or temporary discontinuation and subsequent reintroduction of pirfenidone. For nintedanib, most patients (60%) continued treatment, either at full dose or at a reduced dose, despite a higher frequency of side effects per patient than with pirfenidone. Reassuringly, only 7% of the total number of diarrhoea AEs resulted in discontinuation of treatment with nintedanib.

The discontinuation rate with pirfenidone due to AEs was 29%, which is higher than that seen in the CAPACITY [6] and ASCEND [7] clinical trials. However, this is likely due to the longer duration of treatment equalling four and a half years in our data compared to a one year period in the clinical trials. It is not unexpected that over time that more patients would discontinue treatment due to the combination of disease progression and the cumulative experience of AEs related to pirfenidone treatment. Our data shows that patients experiencing AEs persevere with treatment for up to six months before eventually discontinuing. It was hypothesised that older patients with lower BMIs and more severe disease as depicted by lower FVC and DLCO would be more likely to discontinue treatment due to the occurrence of AEs. However, our data shows that age, BMI and FVC has no impact on pirfenidone tolerability. However patients with more severe disease as denoted by lower DLCO were more likely to discontinue treatment due to AES, however, this was primarily influenced by mortality in this group of patients with lower DLCO.

Our results show that nintedanib was permanently discontinued at a lower rate than identified in the INPULSIS trials, with 19% of our patients permanently discontinued treatment vs. 25.2% in INPULSIS-1 and 23.7% in INPULSIS-2 [10]. This is promising but needs to be interpreted with caution due to our smaller patient cohort and missing data compared to the INPULSIS trials.

Real world patients are often different to those enrolled in clinical trials. For our nintedanib population, some of our patients’ pre-treatment FVC and DLCO values were lower than the inclusion criteria values for the clinical trials. Our treatment population for pirfenidone was older with an average age of 70 years (range 47–91), compared with the ASCEND [7] trial with an average age of 68.4 ± 6.7. Our population also had more severe disease as depicted by lower FVC and DLCO. Sixteen percent of our patients had coexisting emphysema and information from the British Thoracic Society IPF registry [14] of which we contribute 25% of patients demonstrates that real world patients have a high frequency of comorbidities, such as hypertension, ischaemic heart disease and diabetes. The majority of our patients would not have been enrolled into these clinical trials due to the presence of these comorbidities. Despite these differences it is reassuring to find that real world patients have favourable and similar outcomes to those in the clinical trials.

Our results demonstrating a reduction in the decline in FVC after pirfenidone treatment in a real world population over time is important but we accept that our study has some limitations. Our access to lung function results before and after treatment was less than clinical trial patients. Missing data limits the robustness of the analysis, as evidenced by the lower frequency of FVC values at numerous time points. Clinical trials impute missing FVC data when patients die. We have not imputed data for the 20% that have died. Our FVC data is therefore biased towards those patients that survive on treatment and as a result the post pirfenidone FVC values may be biased towards milder patients with higher FVC who are thus more likely to survive. This data highlights how difficult it is to determine efficacy of a therapy within the real world setting.

In terms of managing side effects our early experience with pirfenidone shows that patients who receive regular specialist nurse input to provide support and education improves concordance to treatment [11] therefore it is sensible to follow this model for patients prescribed nintedanib. Both anti-fibrotic treatments have frequent AEs and prompt education and management advice is important for treatment adherence. Telephone and email communication enhance the management of adverse events providing important access to a specialist nurse to advise on a reduction/interruption of treatment [15]. Proactively managing possible adverse effects can reduce the necessity to discontinue pirfenidone [15] and nintedanib.

Most anti-fibrotic gastrointestinal related adverse events (nausea, decreased appetite and weight loss) can be managed by reinforcing the need to take the tablets separately with and during a meal [15]. Continued gastrointestinal symptoms may require the reduction of anti-fibrotic dose and specifically for pirfenidone a gradual increase by 1 tablet every 1–2 weeks to a tolerable dose. Indigestion should be treated in Idiopathic Pulmonary Fibrosis [2] with a Proton Pump Inhibitor to manage gastrointestinal adverse events [15]. Omeprazole might result in a theoretical change to the pharmacokinetics in pirfenidone and should be initially avoided [16] but can be given to patients taking nintedanib. If nausea persists despite dose adjustment interventions such as an antiemetic can be introduced to provide symptomatic relief in order to continue treatment [17]. The main adverse event of nintedanib is diarrhoea, usually mild to moderate but manageable. Recommendations to manage it include rehydration and antidiarrheal medication [18]. If diarrhoea continues a dose reduction may be appropriate and rarely patients have stopped treatment due to the severity and unexpectedness of the diarrhoea. It is worth noting that in our patient cohort the incidence of diarrhoea was significantly different compared with the initial clinical studies 24% versus 62% [10].

Preventative advice is given to every patient treated with pirfenidone about photosensitivity, including; avoiding direct sun and light exposures, applying UVA and UVB sunscreen factor 50 regularly and wearing protective clothing, broad brimmed hat and gloves [19]. Treating a photosensitive rash depends upon it severity. Mild to moderate reactions requires a dose reduction for seven days and restarting treatment if the rash resolves. With a severe reaction including a rash that has continued past 7 days, pirfenidone is discontinued for 15 days then slowly re titrated [15]. Occasionally patients become sensitised to pirfenidone and are unable to restart Pirfenidone due to reoccurrence of a rash even at low doses.

## 5. Conclusions

Our retrospective observational data spanning four and a half years with pirfenidone and 18 months treatment with nintedanib demonstrates favourable preservation of lung function, and similar safety profile and discontinuation rates to previous published data [20]. This is more compelling as our population is older, has more severe disease and significantly more comorbidities compared to those recruited to the clinical trials. Our data provides further ongoing evidence regarding the safety and tolerability of anti-fibrotic therapies for IPF in a real world setting.

## Figures and Tables

**Table 1:** Frequency of Adverse Events with Pirfenidone compared to CAPACITY and ASCEND Clinical Trials.

Adverse Event (AE)	Reported Events	Percentage of Total Events	Percentage of Patients Experiencing AE from CAPACITY [6]	Percentage of Patients Experiencing AE from ASCEND [7]
Appetite Loss	165	17%	11%	15.8%
Nausea and Vomiting	142	15%	36% Nausea 14% Vomiting	36% Nausea 12.9% Vomiting
Lethargy	112	12%	7%	20.9%
Chest Infection	102	10%	-	21.9% (URTI)
Rash	65	7%	32%	28.1%
Gastro-Oesophageal Reflux	71	7%	19% dyspepsia 10% abdominal distension	11.9% gastro-oesophageal reflux 14.7% Dyspepsia
Death	59	6%	-	4%
Weight Loss	48	5%	8%	12.6%
Abdominal Discomfort	42	4%	-	-
Diarrhoea	34	3%	-	22.3%
Other	30	3%	-	-
Deranged Liver Function Tests	15	2%	-	-
Taste Change	15	2%	-	-
Dizziness	19	2%	18%	17.6
Disease Progression	11	1%	-	-
Insomnia	11	1%	10	11.2
Mood Change	6	1%	-	-
Constipation	6	1%	-	11.5
Headache	10	1%	-	25.9
IPF Exacerbation	6	1%	-	-
Right Ventricular Failure	4	0%	-	-
Total AEs	973			

**Table 2:** Frequency of Adverse Events with Nintedanib Compared to INPULSIS Clinical Trials.

Adverse Event (AE)	Reported Events	Percentage of Total Events	Percentage of Patients Experiencing AE from INPULSIS 1	Percentage of Patients Experiencing AE from INPULSIS 2
Diarrhoea	163	24%	61.5%	63.2%
Nausea	90	13%	22.7%	26.1%
Reduced appetite	65	10%	8.4%	12.8%
Tiredness	60	9%	-	-
GOR	53	8%	-	-
Abdominal pain, bloating and wind	50	7%	-	-
Weight loss	29	4%	8.1%	11.2%
Vomiting	20	3%	12.9%	10.3%
Abnormal elevated liver function tests (LFTs)	19	3%	4.9%	5.2%
Death	17	3%	-	-
Constipation	12	2%	-	-
Nose bleeds, haematemesis, haemoptysis	12	2%	-	-
Cough	10	1%	15.2%	11.6%
Taste disturbance	9	1%	-	-
Headache	7	1%	-	-
Low mood	6	1%	-	-
Aching joints	5	1%	-	-
Chest discomfort	5	1%	-	-
AKI	4	1%	-	-
Abnormal blood count results	3	<1%	-	-
Rash	3	<1%	-	-
Increased dyspnoea	3	<1%	7.1%	8.2%
Sleep disturbance	3	<1%	-	-
Dizziness	2	<1%	-	-
Recurrent urinary tract infections (UTIs)	2	<1%	-	-
Bronchitis	2	<1%	11.7%	9.4%
Myocardial infarction (MI)	2	<1%	1.6%	1.5%
Pruritis	2	<1%	-	-
Other viral/ bacterial infection	2	<1%	-	-
Mouth pain/swelling	2	<1%	-	-
Unknown	2	<1%	-	-
P.E	1	<1%	-	-
Hair loss	1	<1%	-	-
Anosmia	1	<1%	-	-
Thigh/calve ache	1	<1%	-	-
Total AEs	668		-	-

## Abbreviations

The following abbreviations are used in this manuscript:

AE	Adverse Event
BMI	Body Mass Index
DLCO	Carbon Monoxide Diffusing Capacity
FBC	Full Blood Count
FVC	Forced Vital Capacity
IPF	Idiopathic Pulmonary Fibrosis
LFTs	Liver Function Tests
NICE	National Institute for Health and Care Excellence
NPP	Named Patient Programme
PDGFR	Platelet Derived Growth Factor Receptor
PFTs	Pulmonary Function Tests
PIN	Patient in Need Programme
UEs	Urea and Electrolytes
UVA	Ultraviolet A
UVB	Ultraviolet B
US FDA	United States Food and Drug Administration
VEGF	Vascular Endothelial Growth Factor Receptor
